# Transcriptome analyses reveal the utilization of nitrogen sources and related metabolic mechanisms of *Sporosarcina pasteurii*

**DOI:** 10.1371/journal.pone.0246818

**Published:** 2021-02-09

**Authors:** Di Pei, Zhiming Liu, Wenjian Wu, Biru Hu

**Affiliations:** 1 College of Liberal Arts and Sciences, National University of Defense Technology, Changsha, Hunan, China; 2 Institute of Chemical Defense, Academy of Military Science, Beijing, China; Guangdong Technion Israel Institute of Technology, CHINA

## Abstract

In recent years, *Sporosarcina pasteurii (S*. *pasteurii)* has become one of the most popular bacteria in microbially induced calcium carbonate precipitation (MICP). Various applications have been developed based on the efficient urease that can induce the precipitation of calcium carbonate. However, the metabolic mechanism related to biomineralization of *S*. *pasteurii* has not been clearly elucidated. The process of bacterial culture and biomineralization consumes a large amount of urea or ammonium salts, which are usually used as agricultural fertilizers, not to mention probable environmental pollutions caused by the excessive use of these raw materials. Therefore, it is urgent to reveal the mechanism of nitrogen utilization and metabolism of *S*. *pasteurii*. In this paper, we compared the growth and gene expression of *S*. *pasteurii* under three different culture conditions through transcriptome analyses. GO and KEGG analyses revealed that both ammonium and urea were direct nitrogen sources of *S*. *pasteurii*, and the bacteria could not grow normally in the absence of ammonium or urea. To the best of our knowledge, this paper is the first one to reveal the nitrogen utilization mechanism of *S*. *pasteurii* through transcriptome methods. Furthermore, the presence of ammonium might promote the synthesis of intracellular ATP and enhance the motility of the bacteria. There should be an ATP synthesis mechanism associated with urea hydrolysis catalyzed by urease in *S*. *pasteurii*.

## Introduction

Microbially induced calcium carbonate precipitation (MICP) refers to the formation of calcium carbonate precipitation by microbial metabolic activities including urea decomposition, photosynthesis, denitrification, ammonification, sulfate reduction, and methane oxidation [1, 2]. To date, a variety of microorganisms have been found to achieve MICP by hydrolysis of urea, and this kind of biomineralization has been used in a wide range of applications, including repairing cracks in buildings and roads, curing sands, making self-healing cements, and developing biomineralized materials [3–11]. Among the urease-producing bacteria, *Sporosarcina pasteurii* (*S*. *pasteurii*) is one of the most common and widely used species in MICP [12].

*S*. *pasteurii* can produce urease, which catalyzes the hydrolysis of urea into carbonate ions and ammonium, and the resulting carbonate ions can react with extracellular calcium ions to form calcium carbonate precipitation [1, 13]. Although this mineralization has been used in a variety of applications, it still has some limitations, including its adaptability to different environmental conditions, uncontrolled bacterial growth or urease activity, instability in large-scale engineering, and the requirement of higher costs than chemical and conventional methods.

Several current studies have attempted to address those issues by optimizing the growth and mineralization conditions of *S*. *pasteurii*. These studies include using response surface methods to optimize the concentrations of urea, calcium chloride and nickel ions to maximize the growth rate and calcium carbonate precipitation capacity of *S*. *pasteurii* [14]. However, it is found that enhanced mineralization alone cannot solve the problems in large-scale engineering applications effectively.

*S*. *pasteurii* has a specific mechanism of nutrient source utilization. It has been shown that *S*. *pasteurii* is unable to utilize glucose [15], and therefore yeast extracts are commonly used in laboratories as its carbon source. Moreover, the efficient urease activity of *S*. *pasteurii* may play an important role in the utilization of nitrogen source, and play a crucial role in the mineralization of *S*. *pasteurii*. Furthermore, it has been reported that the nutritional cost in the culture medium may be as high as 60% of the total operating cost [16]. A large amount of urea and ammonium salts are used in the culture medium, as not only consume a large amount of agricultural fertilizers, but also tend to pollute the environment. Therefore, the high cost of growth nutrients becomes a major obstacle for the commercialization and large-scale application of microbial induced mineralization technology. Under such backgrounds, researchers have begun to work on the development of nutrient alternatives for *S*. *pasteurii*. They have successively adopted human urine and pig urine as alternative nitrogen sources [11, 17, 18], or used industrial wastewater as carbon sources [19, 20], reducing the cost of bacterial mineralization to some degree. However, treatments of raw materials were generally required in those studies, and limited bacterial culture or mineralization are often observed. Therefore, it is both necessary and urgent to investigate the utilization of different nitrogen source of *S*. *pasteurii* and reveal relevant metabolic mechanisms.

In order to get a better understanding of the mechanism of nitrogen source utilization, comparative transcriptome studies are carried out under three different culture conditions on *S*. *pasteurii* in this paper. We aimed to investigate the expression pattern of urease gene of *S*. *pasteurii* and the necessary nutrients for the growth, metabolism, and mineralization of *S*. *pasteurii*. The results presented in the paper can help to reduce the cost of MICP and realize the flexible regulation of mineralization reaction in large-scale engineering applications by the utilization of proper nitrogen sources.

## Methods

### Culture of *S*. *pasteurii*

The *S*. *pasteurii* strain (BNCC 337394) used in this study was purchased from Bnbio (Beina Chuanglian Biotechnology Institute, Beijing). The whole genome sequencing results of this strain were uploaded to NCBI (NZ_CP038012.1) in March 2019 [21]. The received bacterial lyophilized powder was first activated with premade activation medium (tryptone 5g/L, yeast extract 3g/L, urea 20g/L, pH = 7.3) and incubated at 30°C for 24 hours. Then, the obtained small amount of bacterial culture was streaked onto Christensen’s Urea Agar [22] plate and grow at 30°C for 24 hours. A single colony with good growth potential was selected for expansion and subsequent studies. The medium was the NH_4_-YE medium recommended by the American Type Culture Collection (ATCC).

### Growth test and selection of different incubation conditions and times

In order to select suitable culture periods for transcriptome sequencing, the bacteria were cultured for 48 hours in three different medium conditions: (1) control group B (yeast extract 20 g/L); (2) ammonium group N (yeast extract 20 g/L, ammonium chloride 10 g/L); (3) urea group U (yeast extract 20 g/L, urea 5.62 g/L). The urea group and the ammonium group had the same nitrogen contents. The pH of the medium was adjusted to 8.8 using 1mol/L NaOH solution, which is the optimum pH for *S*. *pasteurii*, and the concentration of sodium ion was adjusted using 1mol/L NaCl stock solution to ensure that they were the same in the three groups. Three kinds of 300mL media mentioned above were prepared and stored in 1L conical flasks, after sterilization for 20 minutes under 121°C for subsequent culture usages. The ammonium group and urea group should be prepared by adding solutions of urea or ammonium chloride with filtration-sterilization in advance to the sterilized medium. When culturing, 3mL cell culture (from NH_4_-YE medium) which had an OD_600_ of 1 was centrifuged and inoculated into three different media groups respectively. The OD_600_ of three groups were detected every six hours with UVmini-1240 Spectrophotometer (Shimadzu, Japan). When the OD_600_ of a medium sample was greater than 0.8, it was diluted to ensure its OD_600_ was between 0.2 and 0.8, and the actual OD_600_ of original medium was the reading multiplied by the dilution factor. It was found that both the ammonium group and the urea group showed exponential growth within 24 hours, whereas the control group had limited growth due to lack of nutrients. So the sampling point at 12 hours was chosen, when the growth rate of the bacteria was fastest in the exponential growth stage. Three replicates for each culture condition were sampled.

### RNA extraction and sequencing

Total RNA was extracted from nine sets of bacterial fluid samples using RNAprep Pure Cell/Bacteria Kit (Tiangen Biotech, Beijing), following the manufacturer’s protocol. The quality and quantity of the extracted total RNA were verified using RNA-free agarose gel electrophoresis and Agilent Bioanalyzer 2100 system (Agilent Technologies, Santa Clara, CA, USA). Nine sets of RNA samples were then used for library construction. A total amount of 3μg RNA per sample was used as input material for the RNA sample preparations. As for prokaryotic samples, mRNA was purified from total RNA using probes to remove rRNA. Fragmentation was carried out using divalent cations under elevated temperature in First Strand Synthesis Reaction Buffer (5X). First strand cDNA was synthesized using random hexamer primer and M-MuLV Reverse Transcriptase (RNase H-). Second strand cDNA synthesis was subsequently performed using DNA Polymerase I and RNase H. Remaining overhangs were converted into blunt ends via exonuclease/polymerase activities. After adenylation of 3’ ends of DNA fragments, Adaptor with hairpin loop structure were ligated to prepare for hybridization. Then USER Enzyme was used to degrade the second strand of cDNA containing U. In order to select cDNA fragments of preferentially 370~420bp in length, the library fragments were purified with AMPure XP system (Beckman Coulter, Beverly, USA). Then PCR was performed with Phusion High-Fidelity DNA polymerase, Universal PCR primers and Index (X) Primer. At last, PCR products were purified (AMPure XP system) and the library quality was assessed on the Agilent Bioanalyzer 2100 system.

The clustering of the index-coded samples was performed on a cBot Cluster Generation System using TruSeq PE Cluster Kit v3-cBot-HS (Illumia) according to the manufacturer’s instructions. After cluster generation, the library preparations were sequenced on an Illumina Novaseq platform (Novogene, China) and 150bp paired-end reads were generated.

### Data processing and gene expression level quantification

Raw data (raw reads) of fastq format were firstly processed through in-house perl scripts. In this step, clean data (clean reads) were obtained by removing reads containing adapter, reads containing poly-N and reads of low quality from raw data. At the same time, the Q20, Q30 and GC content of clean data were calculated. All the downstream analyses were based on the clean data with high quality.

Reference genome and gene model annotation files were downloaded from genome website directly. Both building index of reference genome and aligning clean reads to reference genome were used with Bowtie2 v2.3.3.1.

HTSeq v0.6.1 was used to count the reads numbers mapped to each gene. And then FPKM of each gene was calculated based on the length of the gene and reads count mapped to this gene. FPKM (expected number of Fragments Per Kilobase of transcript sequence per Millions base pairs sequenced) considers the effect of sequencing depth and gene length for the reads count at the same time, and is currently the most commonly used method for estimating gene expression levels.

### Differential gene expression analysis

Differential gene expression analysis was performed on the three sequencing data sets (three biological replicates per set) using the DESeq2 R package (1.20.0) [23]. DESeq2 provides statistical routines for determining differential expression in digital gene expression data using a model based on the negative binomial distribution. The resulting P-values were adjusted using the Benjamini and Hochberg’s approach for controlling the false discovery rate (FDR). Genes with an adjusted P-value (PADJ) <0.05 found by DESeq2 were assigned as differentially expressed genes (DEGs). In this study, three replications of group B were taken as control. Compared with the sequencing data of group B, the level of difference shown from that of group N and group U was calculated by using log_2_ (fold change), with the absolute value above 1 indicating DEGs [24], and with the absolute value above 0 indicating up or down regulated expressions of relevant genes.

### GO enrichment analysis and KEGG enrichment analysis

Gene Ontology (GO) enrichment analysis of differentially expressed genes was implemented by the clusterProfiler R package, in which gene length bias was corrected [25]. GO terms with corrected p-value less than 0.05 were considered significantly enriched by DEGs.

KEGG is a database resource for understanding the high-level functions and biological effects of biological systems (*e*.*g*., cells, organisms, and ecosystems) from molecular-level information, especially from large-scale molecular datasets generated by genome sequencing and other high-throughput experimental technologies (http://www.genome.jp/kegg/). ClusterProfiler R package was also used to test the statistical enrichment of DEGs in KEGG pathways [26].

## Results

### Growth test, transcriptome sequencing and data analysis

**Fig 1:**
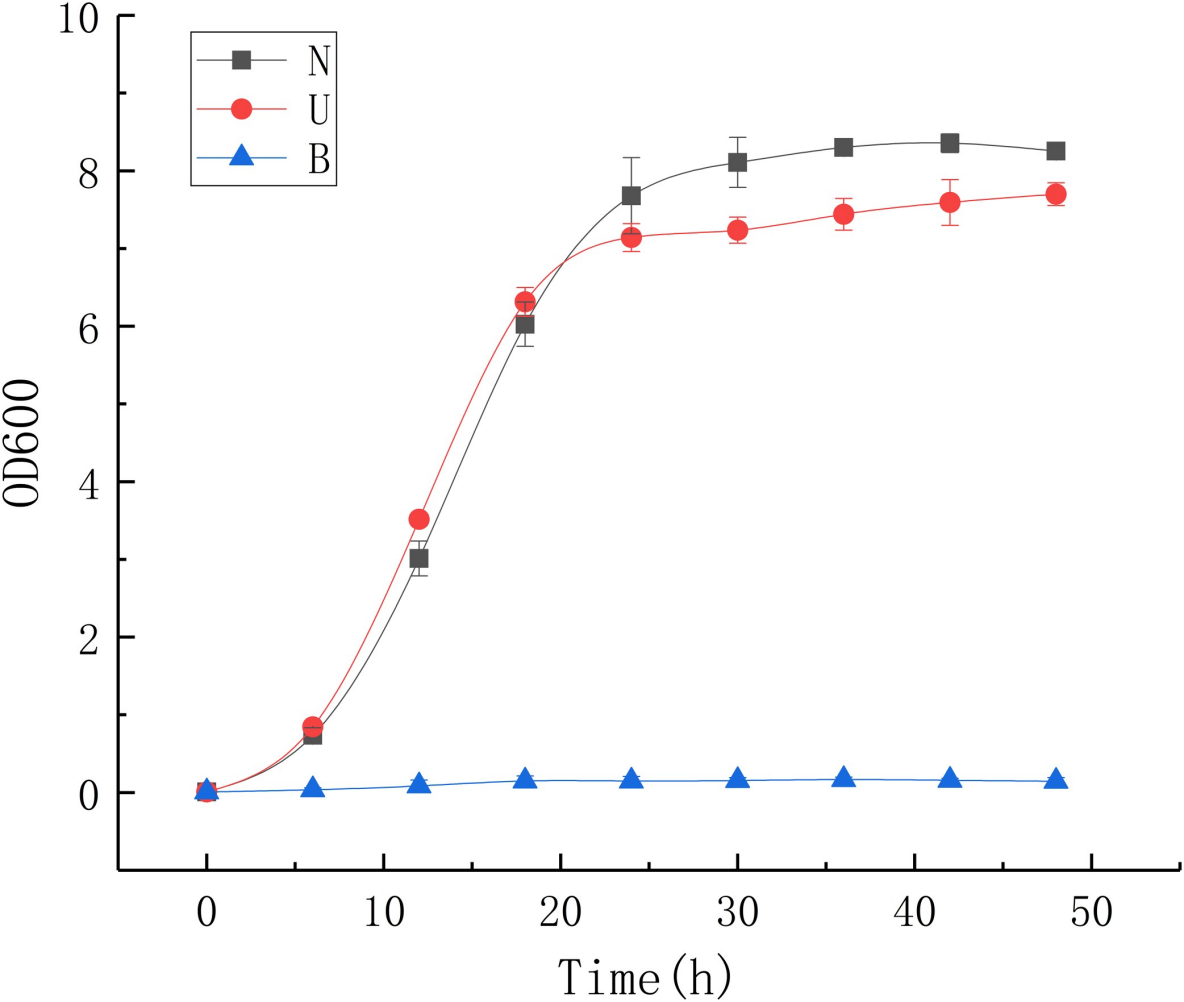
Growth test. The graph is based on the mean and standard deviation of three replicates.

As stated above, the result of growth test showed that both the ammonium group and the urea group had grown exponentially within 24 hours, whereas the control group had limited growth due to lack of nitrogen nutrients (Fig 1).

**Fig 2:**
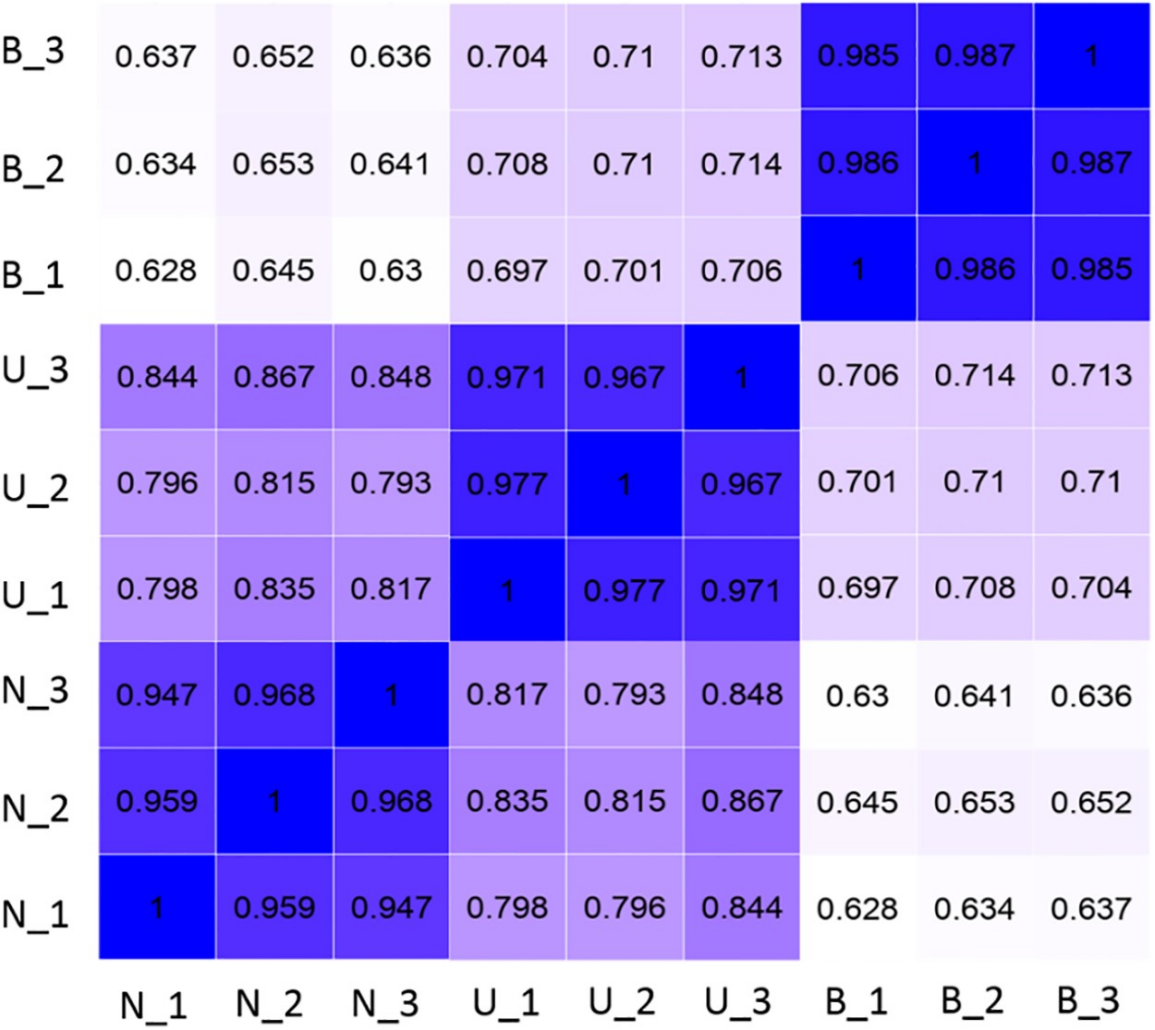
Pearson’s correlation coefficient for the biological repetitions. R^2^: the square of Pearson correlation coefficient.

To study the gene expression differences between *S*. *pasteurii* growth in different media groups and reveal metabolic pathways associated with urease expression, we collected nine bacterial fluid samples (three biological replicates per treatment condition) and constructed complementary DNA (cDNA) libraries. The Pearson’s correlation coefficient was used to confirm the correlation between the three biological replicates in each condition (Fig 2). As expected, the square of the Pearson’s correlation coefficient (R^2^) was greater than 0.86 (Fig 2).

**Fig 3:**
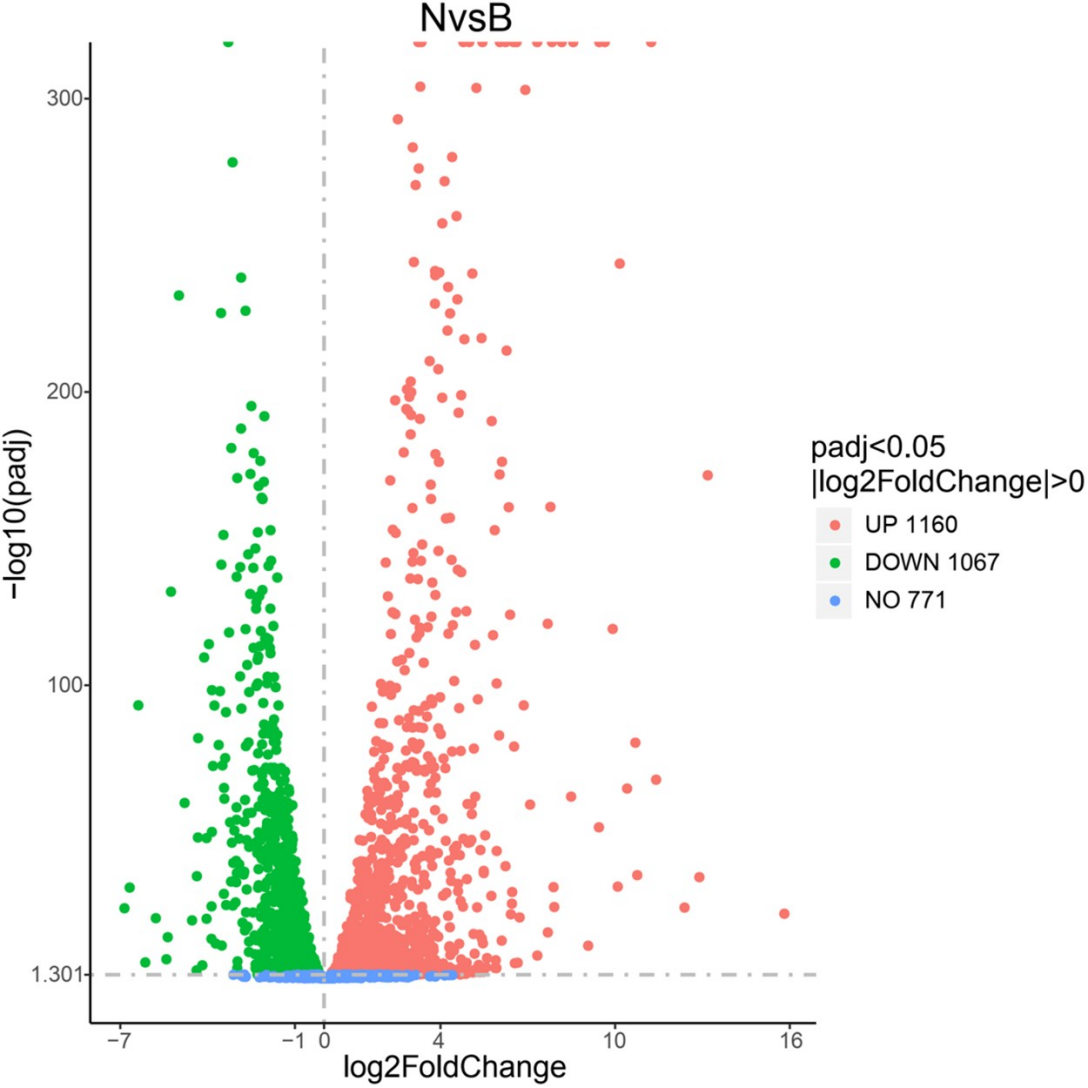
The DEGs’ volcano graph between group N and group B (the control group). The x-axis represents the log_2_(fold change) for each gene of the *S*. *pasteurii* transcriptome. The y-axis represents the negative log_10_ of the FDR-adjusted P-value. Green dots represent genes that were significantly down-regulated. Red dots represent genes that were significantly up-regulated.

**Fig 4:**
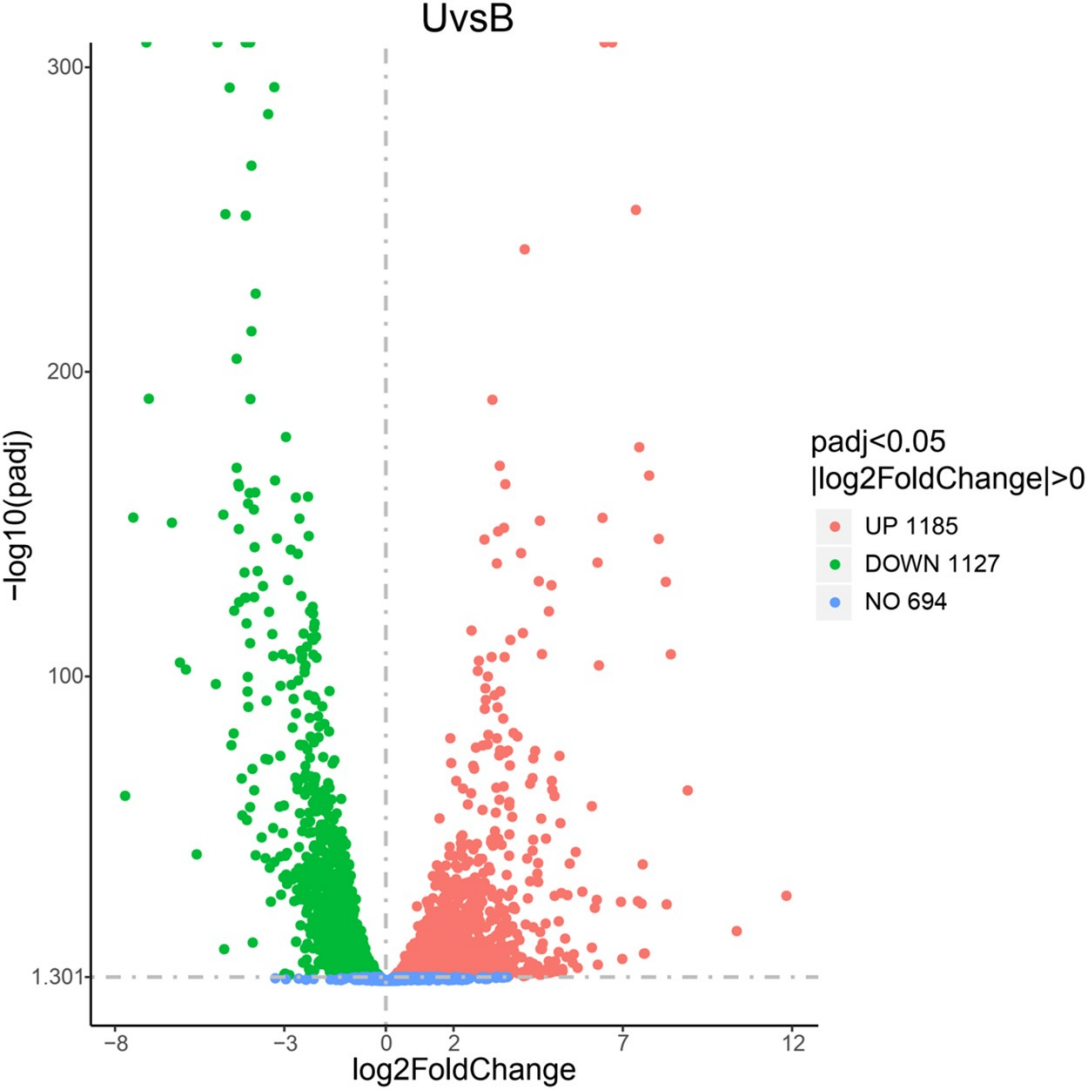
The DEGs’ volcano graph between group U and group B (the control group). The x-axis represents the log_2_(fold change) for each gene of the *S*. *pasteurii* transcriptome. The y-axis represents the negative log_10_ of the FDR-adjusted P-value. Green dots represent genes that were significantly down-regulated. Red dots represent genes that were significantly up-regulated.

**Fig 5:**
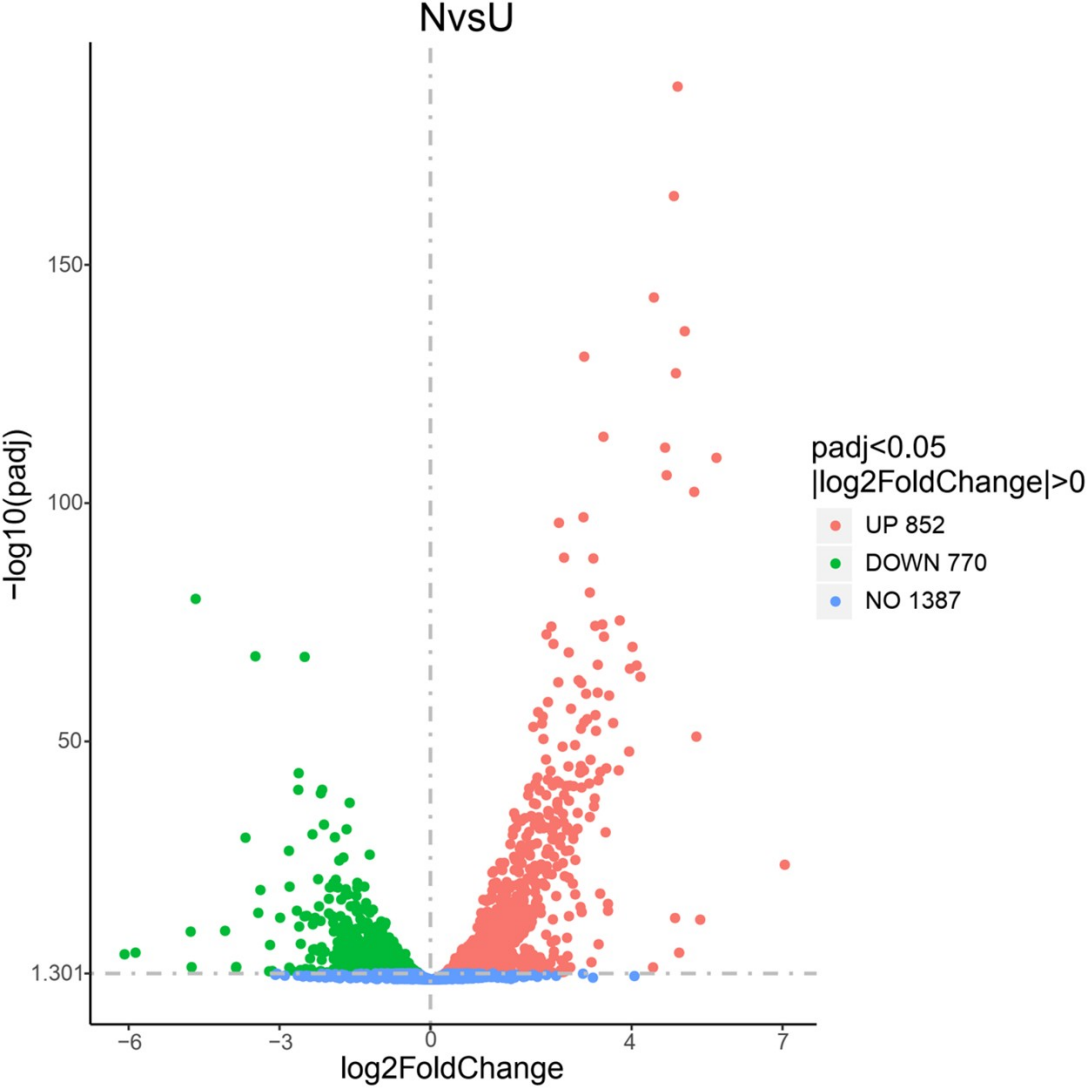
The DEGs’ volcano graph between group N and group U. The x-axis represents the log_2_(fold change) for each gene of the *S*. *pasteurii* transcriptome. The y-axis represents the negative log_10_ of the FDR-adjusted P-value. Green dots represent genes that were significantly down-regulated. Red dots represent genes that were significantly up-regulated.

Based on the genomic data and the data generated from the transcriptome sequencing, a total of 3090 genes were generated and expressed in *S*. *pasteurii* in the experiment (S1 Table). There were 2227 DEGs between the ammonium group and the control group (Fig 3) and 2312 DEGs between the urea group and the control group (Fig 4). The number of DEGs was more than two thirds of the total number of genes, *i*.*e*., both produced large physiological and metabolic differences. Comparing the urea group with the ammonium group, 1622 DEGs were found between the two groups (Fig 5), *i*.*e*., there were a large number of metabolic differences between the two groups. The raw data had been deposited at the Short Read Archive (NCBI) under accession number PRJNA664251.

### Transcriptome expression changes of *S*. *pasteurii* in the absence of ammonium or urea

**Fig 6:**
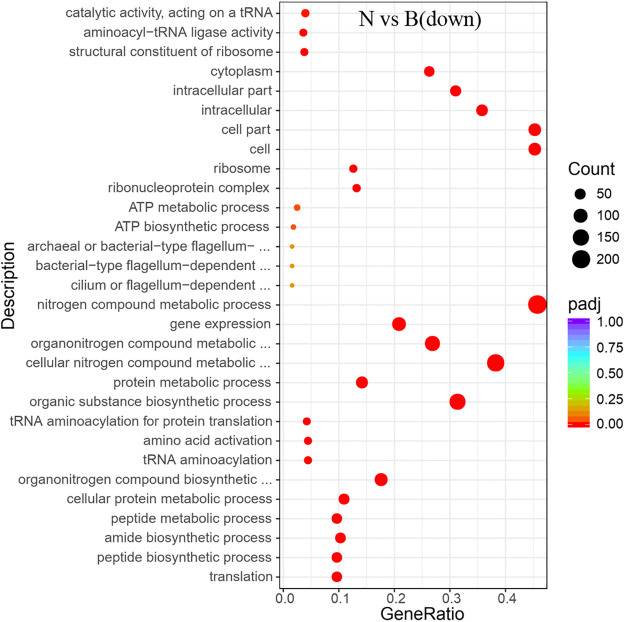
GO enrichment of the transcriptional down-regulated genes in *S*. *pasteurii* in the presence of ammonium. The x-axis represents the ratio of the number of differential genes annotated to the pathway number to the total number of differential genes. The y-axis represents the name of the enriched pathway.

**Fig 7:**
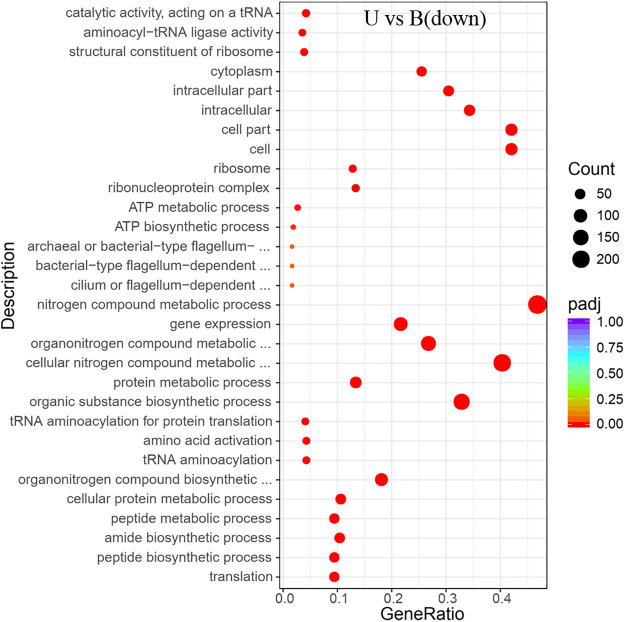
GO enrichment of the transcriptional down-regulated genes in *S*. *pasteurii* in the presence of urea. The x-axis represents the ratio of the number of differential genes annotated to the pathway number to the total number of differential genes. The y-axis represents the name of the enriched pathway.

**Fig 8:**
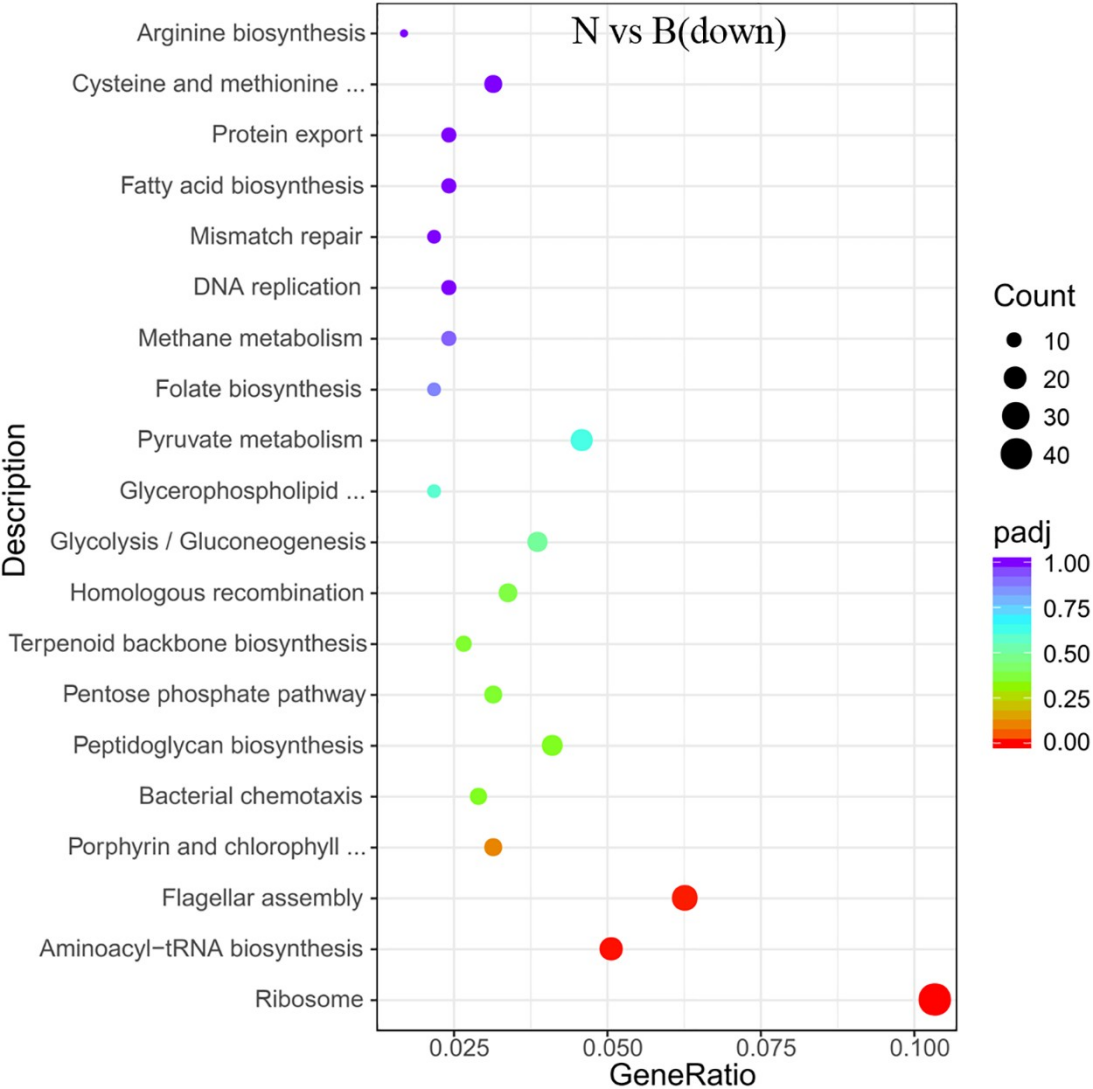
KEGG enrichment of the transcriptional down-regulated genes in *S*. *pasteurii* in the presence of ammonium. The x-axis represents the ratio of the number of differential genes annotated to the pathway number to the total number of differential genes. The y-axis represents the name of the enriched pathway.

**Fig 9:**
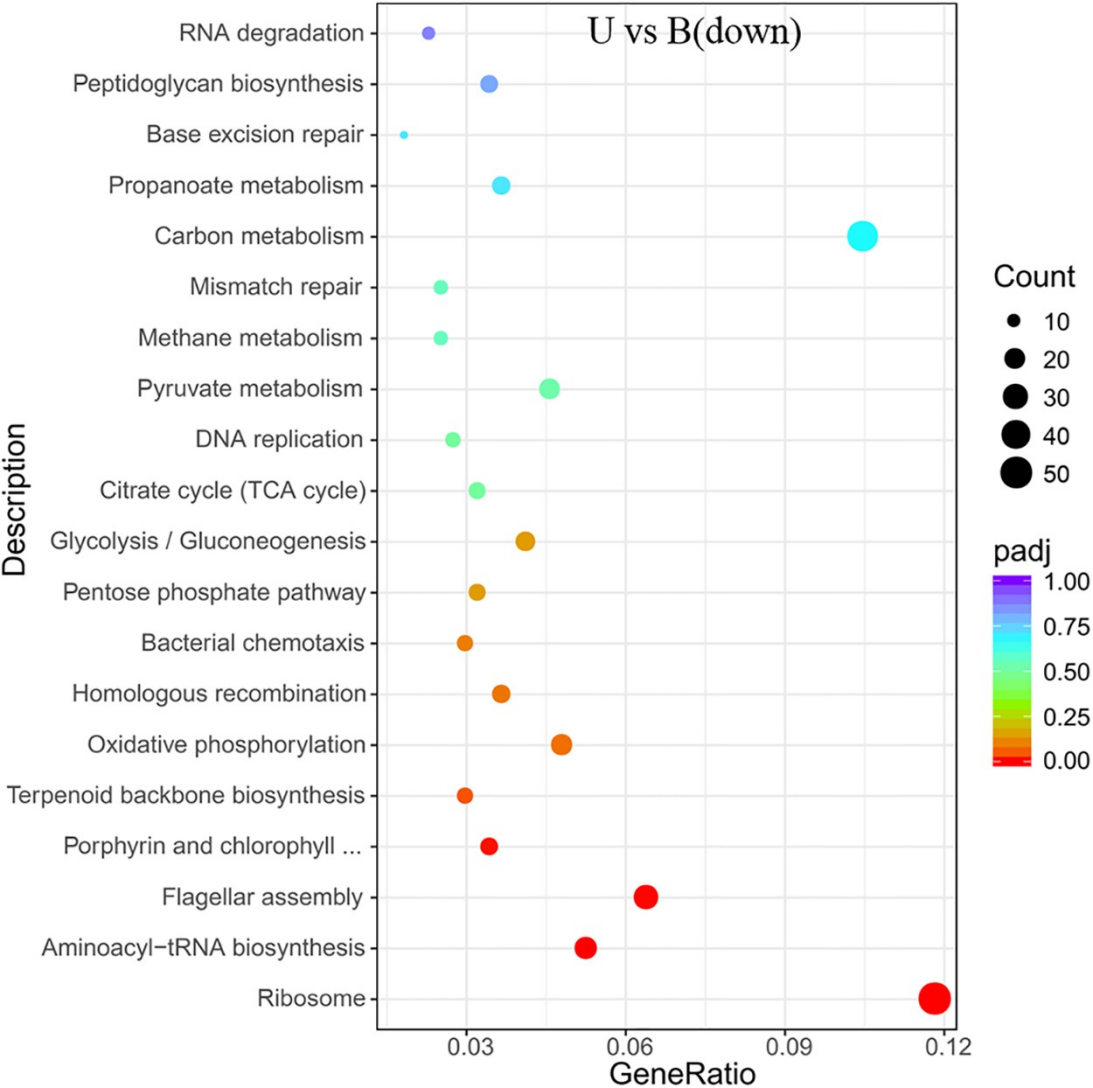
KEGG enrichment of the transcriptional down-regulated genes in *S*. *pasteurii* in the presence of urea. The x-axis represents the ratio of the number of differential genes annotated to the pathway number to the total number of differential genes. The y-axis represents the name of the enriched pathway.

Items that were significantly enriched in the GO analyses were screened out. It was found that the functional proteins involved in the process of gene expression (GO: 0010467) were enhanced fundamentally in the absence of ammonium and urea (Figs 6 and 7). Since transcription was one of the most basic and direct processes in gene expression, the process of gene expression might involve those functional proteins associated with transcriptional activity. After transcription, the genetic information of the mRNA would be expressed as functional proteins through the process of translation. According to the results of GO analyses, it was clear that the expression of functional proteins associated with translation (GO: 0006412) were significantly enhanced, together with the ribosome (GO: 0005840) which was the basic organelles of translation and closely related to translation. The expression of functional proteins of ribonucleoprotein complex and the structural constituents of ribosome (GO: 1990904, GO: 0003735) were similarly significantly enhanced (Figs 6 and 7), and the metabolic pathways of ribosomes showed significant enrichments in KEGG analyses too (Figs 8 and 9). It was known that peptides were obtained after translation and before the formation of mature proteins. The expression of functional proteins related to peptide biosynthetic process (GO: 0043043) was also enhanced. Meanwhile, tRNAs were required to exercise their transport functions by activating and binding the corresponding amino acids during the translation process. Meanwhile, the tRNA aminoacylation for protein translation (GO: 0006418) and amino acid activation (GO: 0043038) of related proteins also showed significant enhancement of expression.

**Fig 10:**
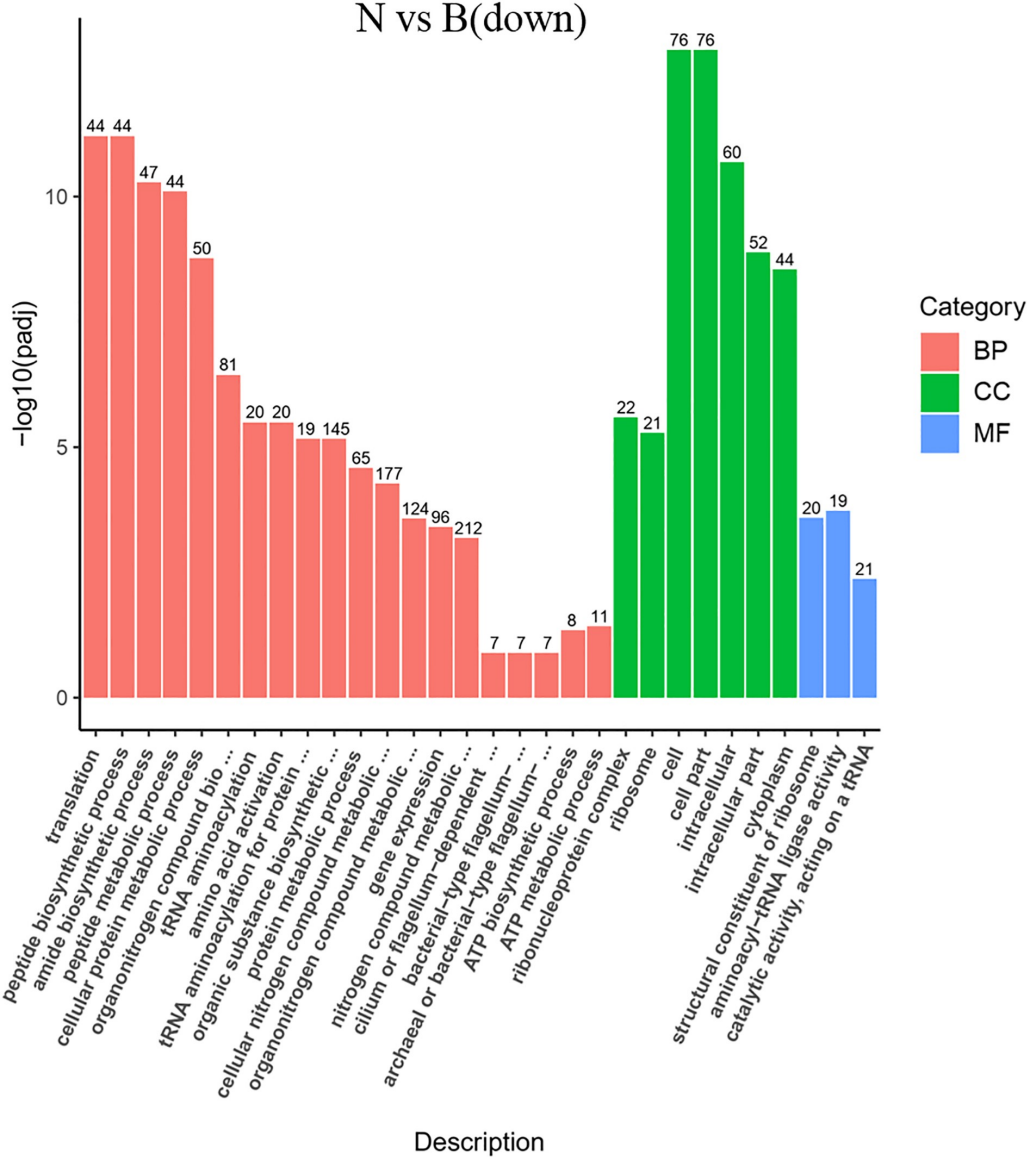
The gene numbers of each enrichment in GO enrichment analysis in the presence of ammonium. Each bar represents the mean (± standard error) of three biological replicates. The x-axis represents the name of the enriched pathway. The y-axis represents the negative log_10_ of the FDR-adjusted P-value.

**Fig 11:**
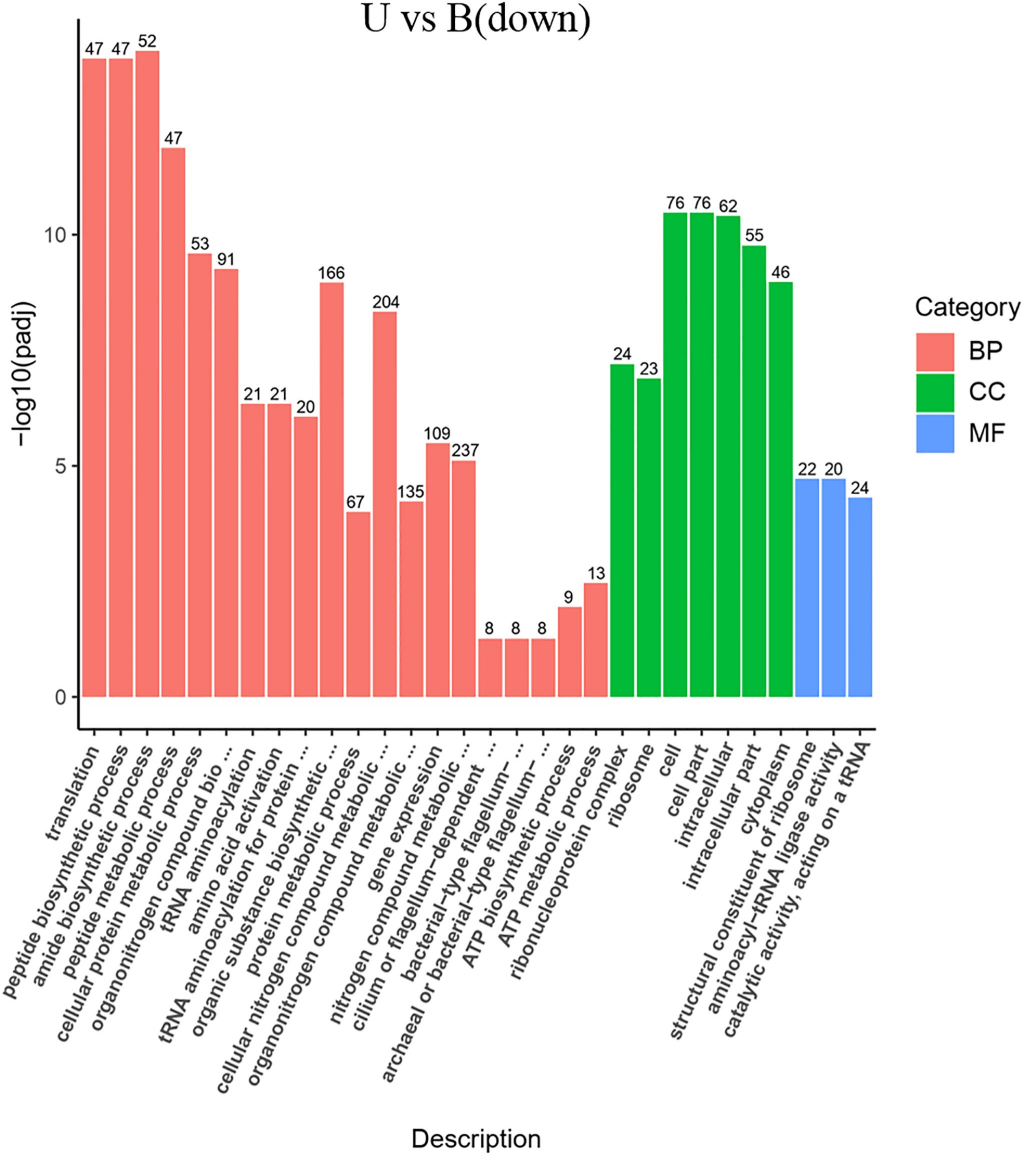
The gene numbers of each enrichment in GO enrichment analysis in the presence of urea. Each bar represents the mean (± standard error) of three biological replicates. The x-axis represents the name of the enriched pathway. The y-axis represents the negative log_10_ of the FDR-adjusted P-value.

**Fig 12:**
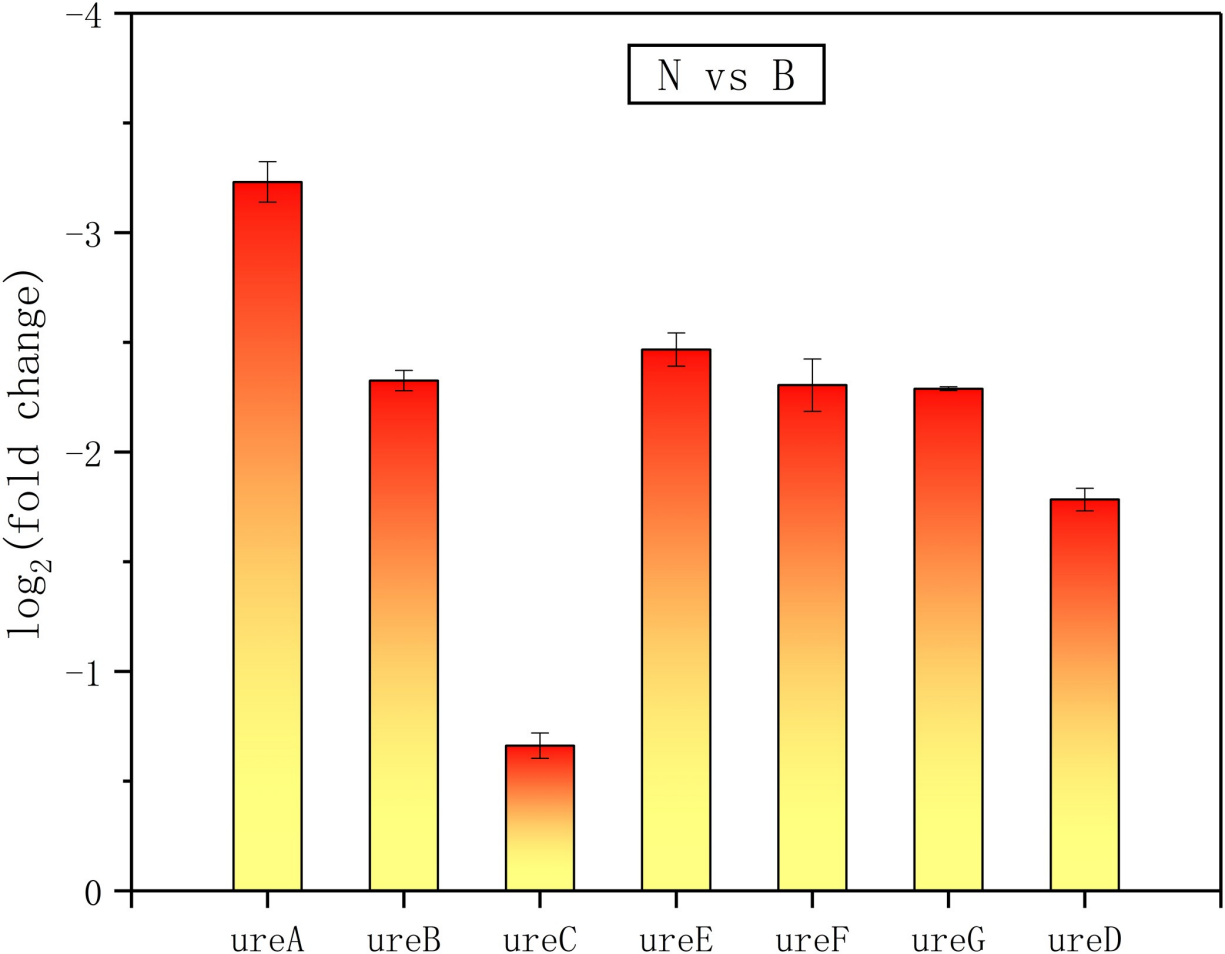
The urease genes differentially expressed in the absence of ammonium. ureA, B, C, D, E and F represent the different subunits and accessory proteins of urease.

**Fig 13:**
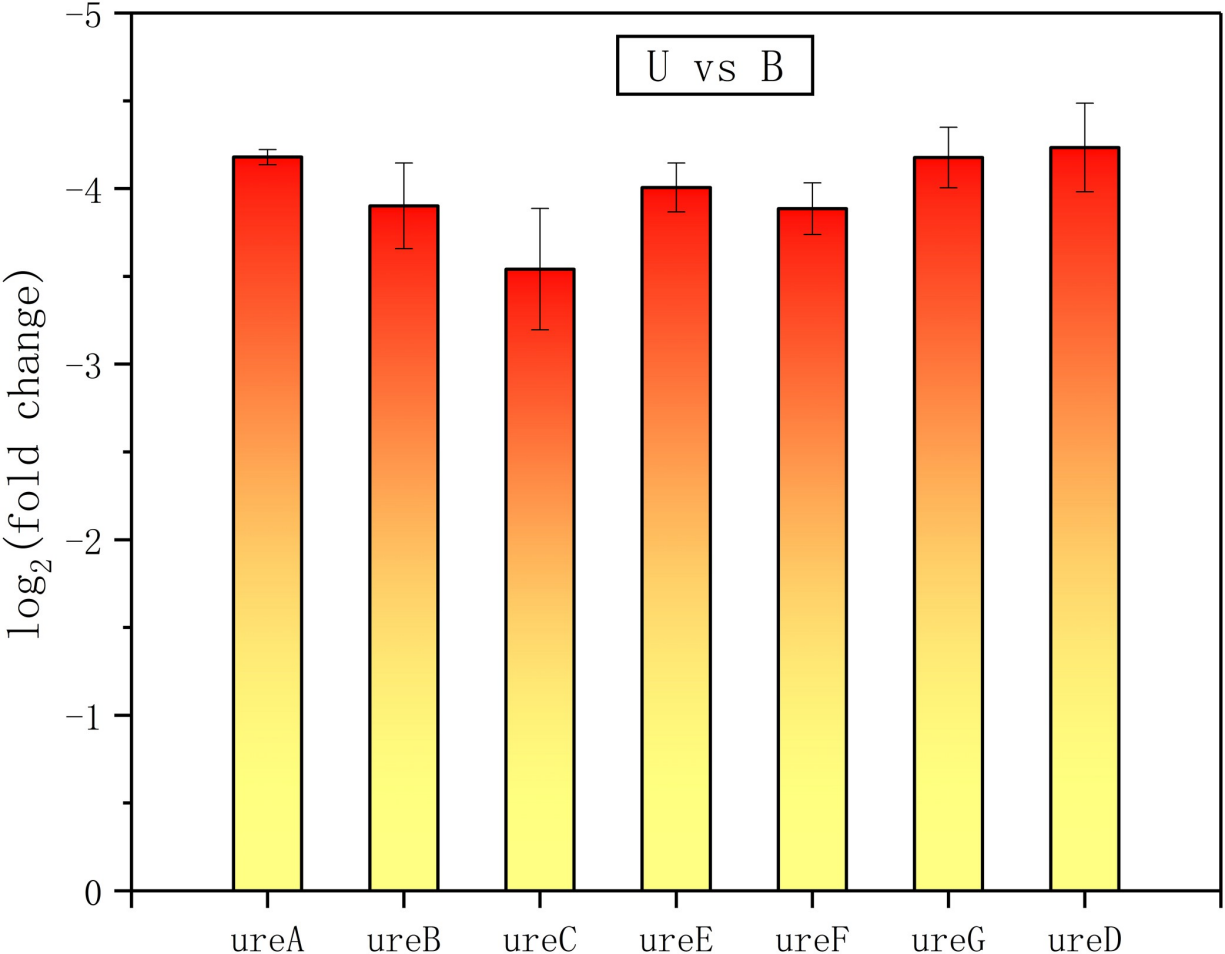
The urease genes differentially expressed in the absence of urea. ureA, B, C, D, E and F represent the different subunits and accessory proteins of urease.

In addition, there were a number of pathways that require the utilization of nitrogen showing enhanced expressions of related functional proteins, such as amide biosynthetic process (GO: 0043604), organic substance biosynthetic process (GO: 1901576), and organonitrogen compound biosynthetic process (GO: 1901566) (Figs 6, 7, 10 and 11). It was noteworthy that numerous functional genes related to the digestion and absorption of nitrogen-containing substances were also enhanced, including peptide metabolic process (GO: 0006518), cellular protein metabolic process (GO: 0044267), protein metabolic process (GO: 0019538), cellular nitrogen compound metabolic process (GO: 0034641), and so on. Among them, what attracted us most was the expression change of urease. Given the function of urease, its associated seven genes were involved in three types of metabolic processes, cellular nitrogen compound metabolic process (GO: 0034641), nitrogen compound metabolic process (GO: 0006807) and organonitrogen compound metabolic process (GO: 1901564). As with the overall changes in those three metabolic processes, all urease genes showed enhanced expressions in the absence of nitrogen sources (Figs 12 and 13). Since the urea hydrolysis by urease was likely to be the main way to obtain nitrogen under natural conditions for *S*. *pasteurii*, ammonium should be a direct nitrogen source for *S*. *pasteurii* under laboratory conditions.

It was believed that most of the differences in the control group without ammonium or urea could be attributed to the restricted growth of bacteria because of lacking nitrogen sources compared with the other two groups, so the overall differences were especially focused on nitrogen utilization related metabolic pathways.

### Differences in cell wall and flagellum gene expression in the presence or absence of nitrogen sources

The cell wall of prokaryotic bacteria is mainly composed of peptidoglycans, however, the cell walls of Gram-positive and Gram-negative bacteria contain their own unique components [27]. The cell wall of Gram-positive bacteria consists of about 90% peptidoglycan and 10% lipoteichoic acid in weight. It had been shown above that the metabolism of *S*. *pasteurii* regarding peptide biosynthesis was enhanced in the absence of a nitrogen source, and the synthesis of peptidoglycans might be associated with that metabolism. As to the synthesis of lipoteichoic acid, it was found that the gene (E2C16_RS11295) (S2 Table) associated with the expression and transport of lipoteichoic acid was similarly enhanced in the absence of a nitrogen source.

One of the most important things about bacterial cell walls is that bacteria have flagellum structures, and the presence of this structure endows them the ability to move and gather to nutrients or escape from harmful substances [28]. Then, in the absence of a nitrogen source, bacteria need greater motility to alleviate nutrient deprivation. In accord with this, the GO analyses revealed that genes related to bacterial-type flagellum-dependent cell motility (GO: 0071973) functions were all markedly up-regulated (Figs 6 and 7). Two thirds of the 39 genes of *S*. *pasteurii* on flagellum assembly and composition showed enhanced expressions. Specifically, 26 flagellum-related genes were up-regulated without the nitrogen source in contrast to the ammonium-containing case (S1 Fig). In contrast to the urea-containing case, 28 flagellum-related genes were up-regulated without nitrogen sources (S2 Fig) (S2 Table). Among these genes, it was known that FliD and FliC were related to the flagella filament that acted as a helical screw to produce thrust for swimming motility. FliN, FliM and FliG were responsible for the C ring structure acting not only as a central part of the rotor for torque generation but also as a structural device to switch the direction of motor rotation. FliP, FliO, FliI and FliH constituted the type III protein export apparatus that was responsible for transporting axial component proteins from the cytoplasm to the distal end of the growing flagella structure to construct the axial structure beyond the cellular membranes. FliK was related to hook structures to control the hook length and FlgG was related to the distal rod. Both FliK and FlgG were involved in regulating the bending flexibility of the hook and rod structure [29, 30]. The increased expression of these genes might allow for enhanced motility of *S*. *pasteurii* to approach the nutrients to survive the nitrogen starvation [31].

There was also a significant enrichment in KEGG about flagellum assembly pathway (Figs 8 and 9) in both the ammonium-containing case and the urea-containing case. Those results were in accordance with the research published in 2020 about the differences in flagellum gene expressions [21].

### Changes in the expression of ATP-biosynthesis related proteins

**Fig 14:**
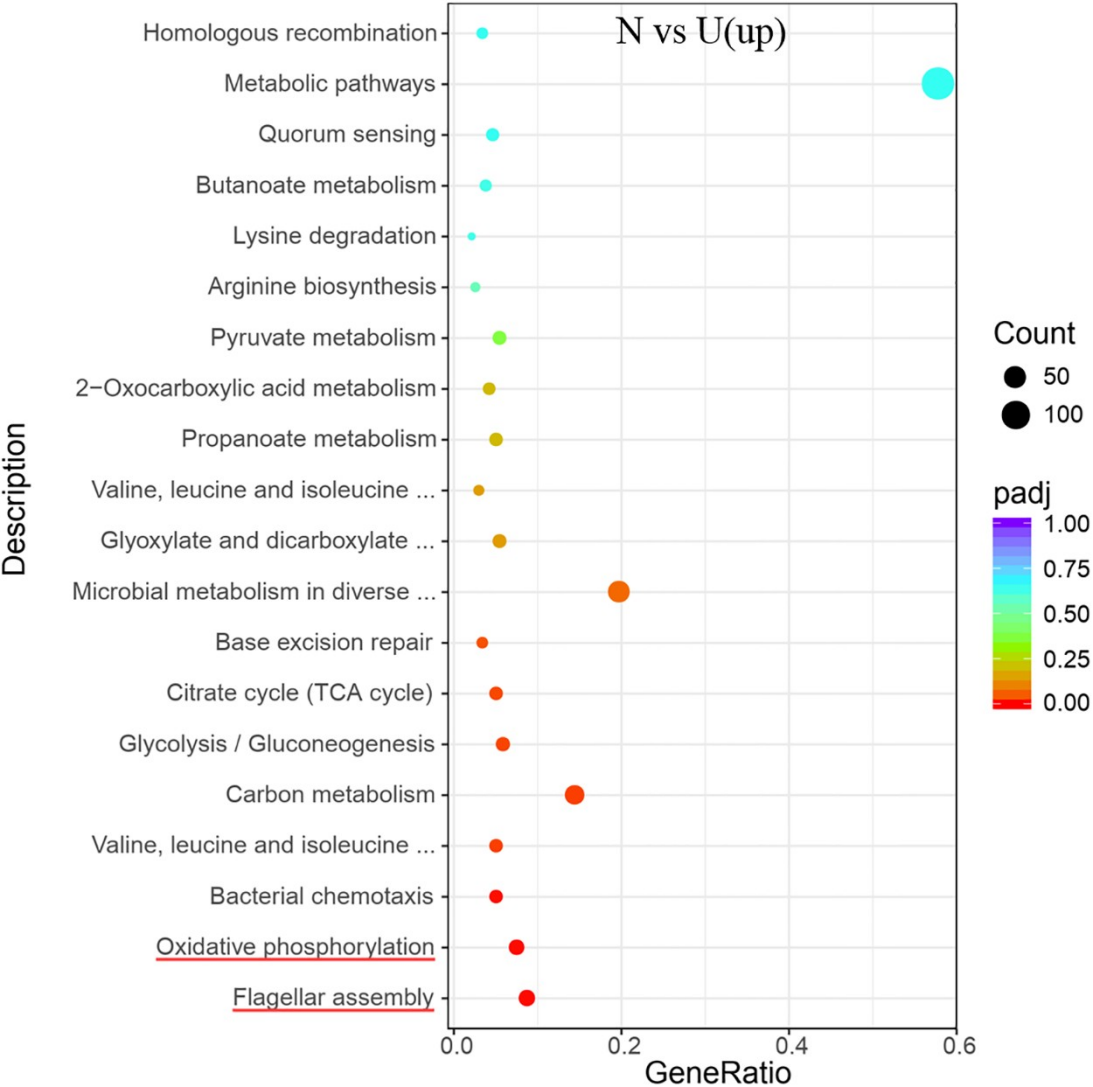
KEGG enrichment of the transcriptional up-regulated genes in *S*. *pasteurii* between groups of N and U. The x-axis represents the ratio of the number of differential genes annotated to the pathway number to the total number of differential genes. The y-axis represents the name of the enriched pathway.

Comparison of the ammonium and urea groups revealed that the DEGs of the two groups showed significant enrichment in the oxidative phosphorylation metabolic pathway in the KEGG analyses (Fig 14). Based on the previous inference that urea or ammonium was the nitrogen source of *S*. *pasteurii*, the difference between the two groups with different nitrogen sources mainly lied in whether the urease expressed by the bacteria was required to catalyze urea hydrolysis, *i*.*e*., whether there was a urea catabolic reaction. The physiological differences caused by the urease hydrolysis reaction in *S*. *pasteurii* was obvious when comparing the differences between these two groups, revealing the physiological significance of urease hydrolysis reaction for *S*. *pasteurii*. The results indicated that the metabolic pathway of oxidative phosphorylation was significantly enriched in the KEGG analysis. As for *S*. *pasteurii*, since the oxidative phosphorylation was a coupling reaction between ADP and inorganic phosphate for the synthesis of ATP, the energy released during the substance oxidations in the cytoplasm of *S*. *pasteurii* was transferred through the electron transfer chain to it, and about 95% of the ATP in the organism came from this way [30].

**Fig 15:**
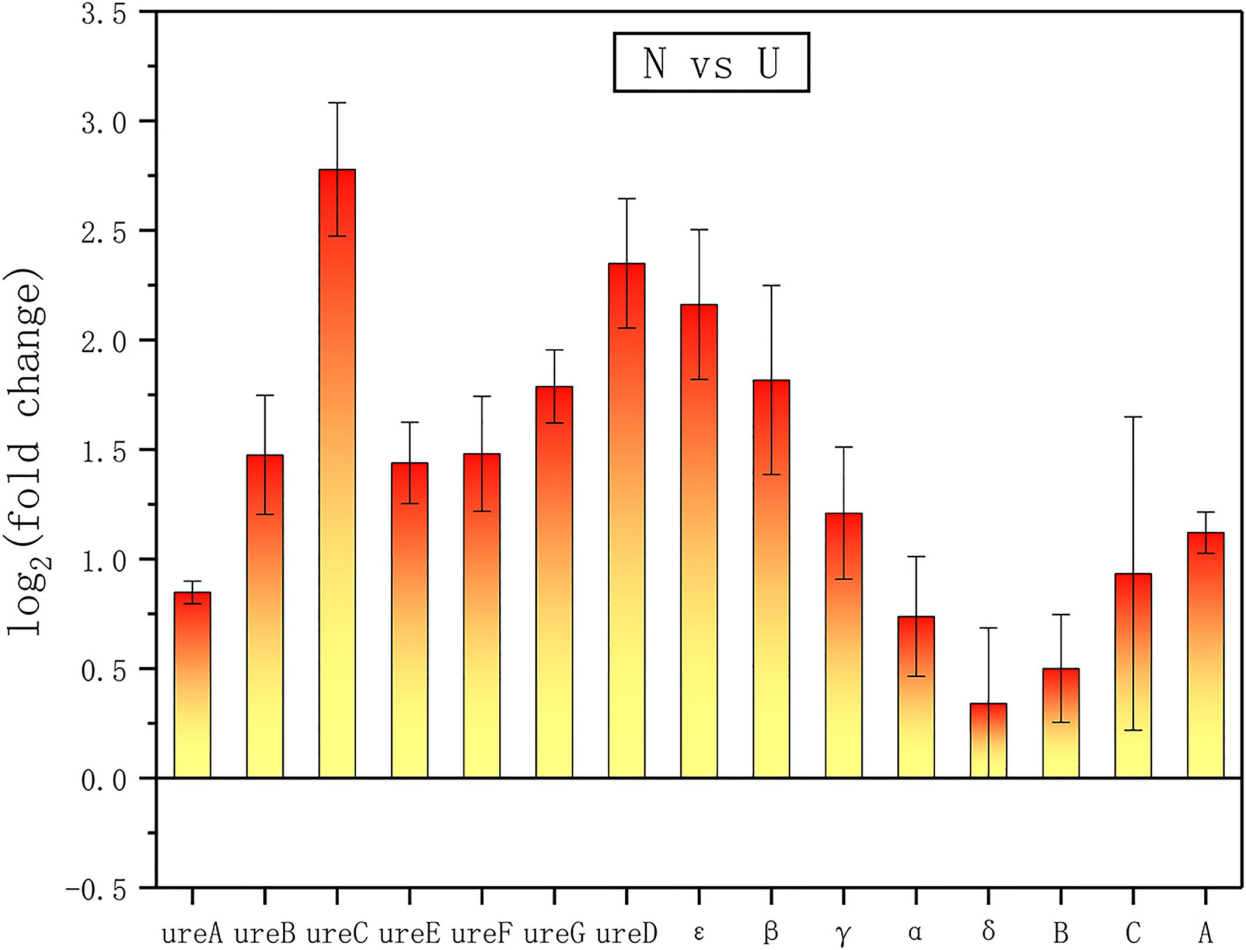
The differentially expressed urease and ATP synthase genes between groups of N and U. urea, B, C, D, E and F represent the different subunits and accessory proteins of urease. α, β, γ, δ, ε, A, B and C represent the different subunits of F0F1 ATP synthase.

**Fig 16:**
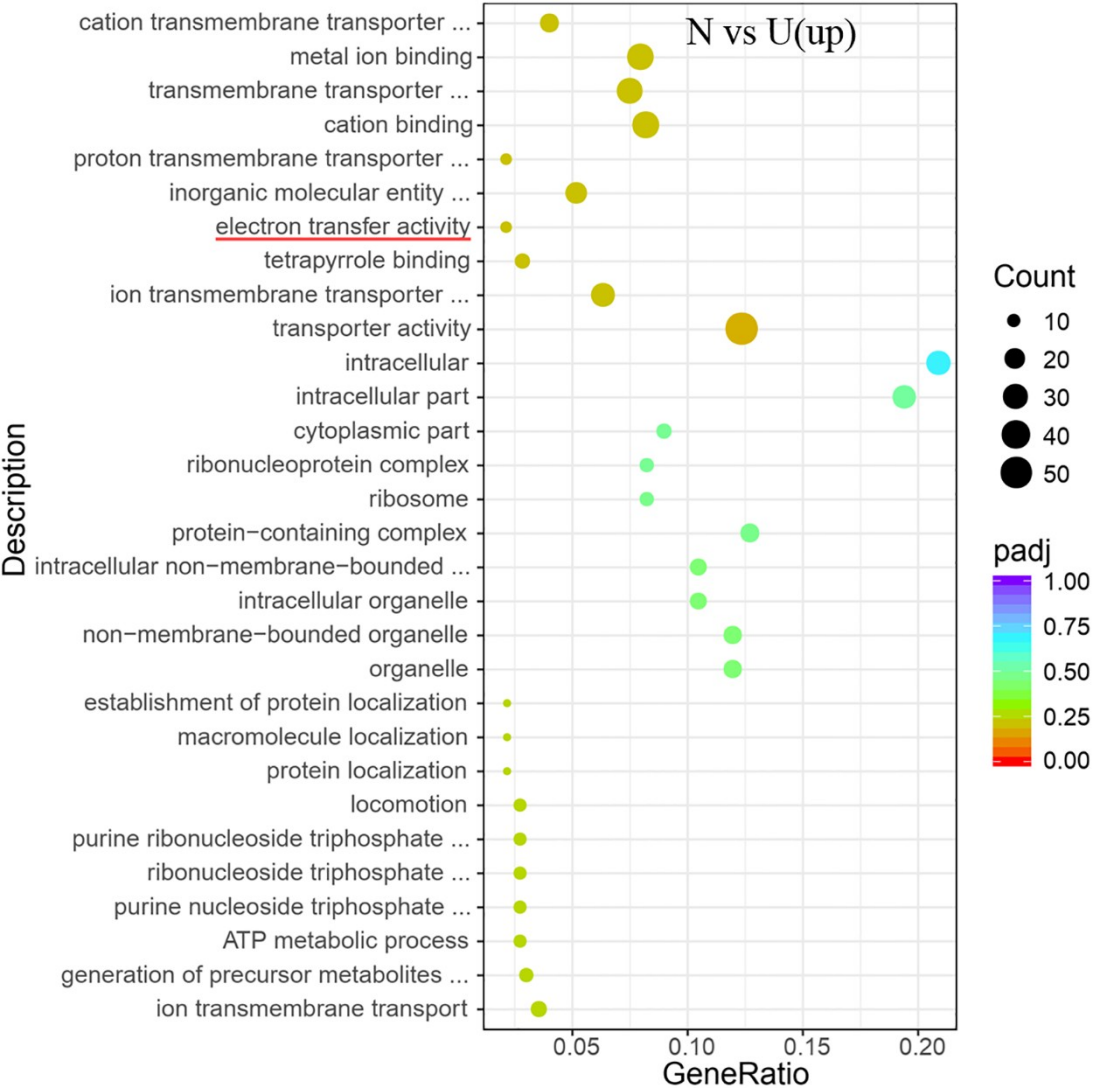
Go enrichment of the transcriptional up-regulated genes in *S*. *pasteurii* between groups of N and U. The x-axis represents the ratio of the number of differential genes annotated to the pathway number to the total number of differential genes. The y-axis represents the name of the enriched pathway.

The data showed that ATP synthase and urease were markedly up-regulated in the ammonium group (Fig 15), and the bacteria grew normally in both groups. The GO analyses showed that the electron transfer activity was significantly higher in the ammonium group than it was in the urea group (Fig 16). Jahns *et al*. showed that *S*. *pasteurii* might have an ATP production mechanism associated with urea catabolism [32]. Once the bacteria of *S*. *pasteurii* were under hypoxic environments, the synthesis of urease in the bacteria would stop [33]. It was known that *S*. *pasteurii* was unable to utilize glucose. Therefore, the metabolism of oxygen in the bacteria was probably to be oxidative phosphorylation which was closely related to the synthesis of ATP. From this perspective, the synthesis of urease might require the support of ATP, *i*.*e*., the synthesis of urease required an increase in oxidative phosphorylation activity indirectly. As an energy currency, ATP was quickly exhausted during hypoxia, leading to the termination of urease synthesis. Moreover, the ammonium was involved in the regulation of the internal pH of the bacteria and leaded to an increase in the bacterial proton electric potential, which stimulated the synthesis of ATP [32]. It was consistent with the results of Jahns’s that the addition of ammonium could stimulate ATP synthesis more directly and efficiently compared to the addition of urea at the same nitrogen content. Such a result might be due to the fact that ammonium was a direct factor in stimulating ATP synthesis in *S*. *pasteurii*. However, when urea was present in the environment, the bacteria’s efficient urease could catalyze urea hydrolysis to the ammonium required for its metabolism, thus completing the ATP synthesis and other metabolic activities.

Taken together, the above results indicated that the ammonium group had an advantage over the urea group in the energy production of *S*. *pasteurii*. In addition, the KEGG analyses of the two differentially expressed genes showed significant enrichment in flagellum assembly (Fig 14). The advantage of ammonium group in energy production enabled it to supply more flagellum movement, thus providing the cells with greater motility.

## Discussion

Overall, the expression of those functional proteins utilizing nitrogen in the *S*. *pasteurii* genome was significantly enhanced in the absence of ammonium or urea. It was found that the transcription and translation of the entire gene expression pathway were severely affected. The metabolism and synthesis of amino acids, polypeptides and proteins were also significantly affected. The above-mentioned genes were all down-regulated when the nitrogen source was sufficient, indicating that the bacterial cells did not need so many genes to be activated to perform these functions when the nitrogen source was sufficient. Expressions of these genes and the proteins that maintained the basic life activities of the bacteria would be enhanced only if lacking the corresponding nutrient sources, and the accessing activities to the likely nutrient source would be strengthened finally.

Although in this paper we pointed out that ammonium and urea were nitrogen sources for *S*. *pasteurii*, it could be seen that the metabolism of large molecules, such as proteins and peptides, were also enhanced in *S*. *pasteurii* in the absence of the above two substances. It was conceivable that amino acids could be directly absorbed and utilized by bacteria. Since *S*. *pasteurii* couldn’t utilize glucose, then amino acids were likely to be taken up and utilized as a carbon source, as was shown by Farrell and Yang *et al*. [34, 35]. Yang *et al*. noted that amino acids could be ammonized by soil bacteria on the cell surface, which was then taken up and utilized [35]. However, our experiment showed that no such metabolic pathway seemed to exist in *S*. *pasteurii*. Instead, in the process of protein synthesis in *S*. *pasteurii*, it was believed that most of the amino acids absorbed externally could be used directly, and ammonium or urea might be used to synthesize other nitrogenous substances or individual amino acids as a nitrogen source.

In addition, some studies had reported that *S*. *pasteurii* lacked an ammonium transport system and its uptake and utilization of ammonium were dependent on alkaline environmental conditions [36], which explained its characteristics as an alkalophilic bacterium. Ammonium also exerted an important influence on intracellular pH in *S*. *pasteurii* [37, 38], either by direct ammonium uptake or by obtaining ammonium through urea hydrolysis, which increased intracellular pH. Since most of the metabolic wastes of living organisms were converted into acids, the metabolic pathway of *S*. *pasteurii* might help it to achieve intracellular pH regulation, and therefore the existence of ammonium or urea had multiple uses and effects on *S*. *pasteurii*.

The overall change in the expression of flagellum genes also provided strong evidence for the inference that ammonium and urea were direct nitrogen sources of *S*. *pasteurii*, as the genes encoding constituent proteins of flagellum were significantly up-regulated in KEGG analyses under culture conditions lacking of ammonium or urea. The flagellum was a special structure that grew on the bacterial cell wall, and the presence of this structure endowed the bacteria the ability of movement. The changes in the expression of flagellum genes under experimental conditions almost certainly served to enhance the accessing ability to nutrients. Based on the above phenomena and results, it could be inferred that both ammonium and urea were the direct source of nitrogen nutrients which were prerequisites for the growth of *S*. *pasteurii*.

There were several differences in energy production of *S*. *pasteurii* in the KEGG analyses between groups of N and U, including significant differences in oxidative phosphorylation metabolic pathways, in the ATP synthase expressions, and in the expression of urease genes. Combining the GO analyses results of the groups of N and B, as well as those of the groups of U and B, it was found that the changes in ATP synthesis and metabolism of *S*. *pasteurii* also exhibited in the absence of either ammonium or urea. According to Jahn’s findings that ammonium was directly responsible for stimulating ATP production in *S*. *pasteurii*, our results also suggested that the stimulation of ATP synthesis by ammonium might persist even when urease was inhibited by the inhibitor. So, it was reasonable that urea played a similar role because of the hydrolysis of urea by urease. It was conceivable that the urea hydrolysis was catalyzed by urease, enabling the bacteria to obtain a direct nitrogen source at first, and the ammonium which was a product of urea hydrolysis played a key role in regulating the internal pH of the bacteria, increasing the bacterial electric potential and stimulating ATP synthesis in sequence.

In summary, through the transcriptome analyses, we inferred that ammonium and urea were both direct nitrogen sources for *S*. *pasteurii* and played a crucial role in its energy supply. However, when urea was used as a nitrogen source, the inferred bacterial motility was the poorest in the three sets of our experimental conditions. Therefore, in the applications where the bacteria are required to undergo continuous mineralization in specific locations, choosing ammonium as a nitrogen source for *S*. *pasteurii* would be more effective according to our findings. As it is known that the ideal compositions of culture medium should be in proportion to the bacterial requirements for each element. The choosing of cheap medium and mineralization conditions for *S*. *pasteurii* needs to pay attention to the supply of ammonium or urea. Our study provides reliable references for the refined preparation of culture media in future large-scale applications, thus may save the application costs. The results of our study contribute to the understanding of the function of nitrogen nutrients to *S*. *pasteurii*, and they may also be used to regulate the urease activity or control the growth stages of the bacteria, *etc*.

## Supporting information

S1 Table Genes generated and expressed in *S*. *pasteurii* in this experiment. (XLS)

S2 Table The list of DEGs related to flagella and cell wall in the presence of ammonium or urea. (XLSX)

S1 Fig Differences in flagellum gene expression in the presence of ammonium. The genes with green boxes were significantly down-regulated. Legend represents the log_2_(fold change) of differentially expressed genes.
(TIF)

S2 Fig Differences in flagellum gene expression in the presence of urea. The genes with green boxes were significantly down-regulated. Legend represents the log_2_(fold change) of differentially expressed genes.
(TIF)
