# Supplementary figures and images for: Transcriptome analyses reveal the utilization of nitrogen sources and related metabolic mechanisms of Sporosarcina pasteurii
Source: PLoS One. 2021 Feb 9;16(2):e0246818. doi: 10.1371/journal.pone.0246818 (PMC7872227; doi:10.1371/journal.pone.0246818)

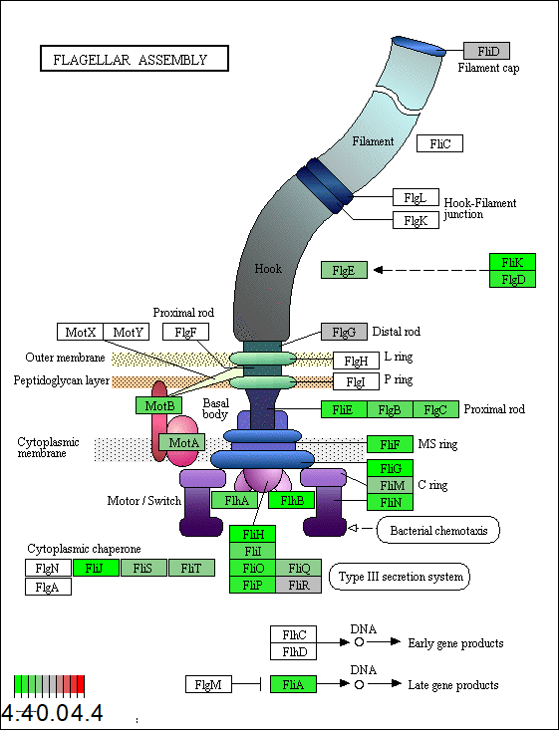

Supplement: S1 Fig — The genes with green boxes were significantly down-regulated. Legend represents the log2(fold change) of differentially expressed genes. (TIF) [file pone.0246818.s003.tif]

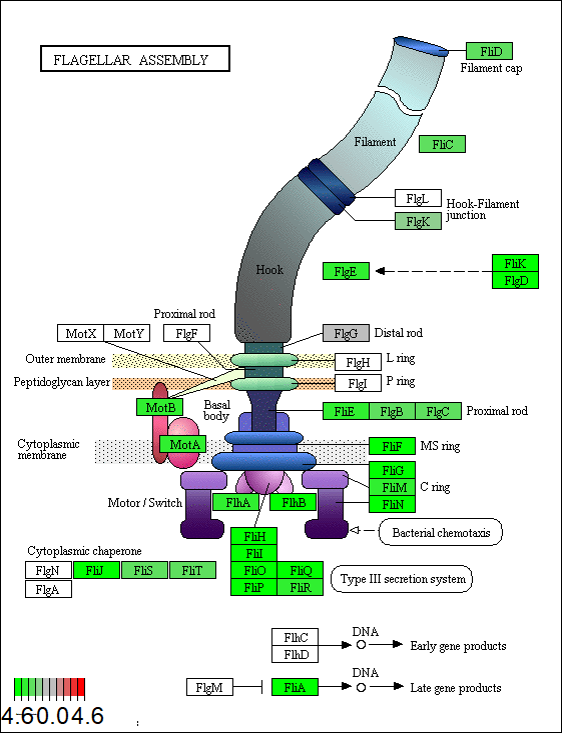

Supplement: S2 Fig — The genes with green boxes were significantly down-regulated. Legend represents the log2(fold change) of differentially expressed genes. (TIF) [file pone.0246818.s004.tif]
